# Why do GPs with a special interest in headache investigate headache presentations with neuroradiology and what do they find?

**DOI:** 10.1007/s10194-011-0375-8

**Published:** 2011-09-29

**Authors:** Steven Elliot, David Kernick

**Affiliations:** 1 Horizon Centre, 94 Littleton Road, Salford, M7 3SE UK; 2 St Thomas Health Centre, Cowick Street, Exeter, EX4 1HJ UK

**Keywords:** Headache, Neuroradiology, Primary care

## Abstract

The general practitioner with a special interest in headache offers an important contribution to the management of headache in primary care where the majority of presentations take place. A number of guidelines have been developed for neuroradiological investigation of headache, but their clinical utility and relevance is not known. Fourteen general practitioners with a special interest in headache recorded consecutive headache consultations over a 3-month period, whether patients were investigated with neuroradiology and if so the reason for investigation and outcome. Reason for investigation was compared to the guidelines published for the use in primary care. 895 patients were seen, of whom 270 (30.1%) were investigated. 47% of indications were outside the guidance framework used, the most common reason for investigation being reassurance. Of those investigated, 5.6% showed positive findings but only 1.9% of findings were felt to be of clinical significance. General practitioners with a special interest investigated with neuroradiology a greater level than general practitioners, but less than neurologists. However, yields of significant findings are broadly comparative across all groups. This report confirms other studies that suggest that even when there is a high level of clinical suspicion, yields of significant findings are very low.

## Background

The majority of headache sufferers are reluctant to seek help and when they do, the condition is often poorly managed by the general practitioner (GP) [1]. In the UK, headache is the most common reason for a secondary care neurological referral but only a small number of neurologists have a special interest in the area and many referrals are inappropriate for a secondary care setting [2].

Reflecting these concerns, it has been suggested that clinics staffed by general practitioners with a special interest (GPwSI) working either in secondary care or in community headache clinics [intermediate care] should support GP colleagues who would continue to provide first-line headache care [3, 4]. A GPwSI is a general practitioner who has developed enhanced skills so as to provide a variety of extended services that has traditionally been provided by secondary care specialists, and training and accreditation frameworks are available in a number of clinical areas including headache [5]. It has been suggested that a GPwSI headache service can satisfy patients with similar headache impact as those seen in secondary care at lower cost [6].

In the UK, the annual primary care consultation rate for headache is 4.4 per 100 patients of which 3% are referred to secondary care [7] where headache accounts for over 20% of new cases [8]. Although a brain tumour can present with a number of symptoms, headache is invariably a cause for concern for both patient and doctor. In the UK, the annual incidence of adult primary brain tumour of patients who present with headache is 0.01% of which 72% will present above the age of 50 [9] and when a patient presents to his/her GP with headache, the risk of a brain tumour is 0.09% [10].

Although a number of headache investigation guidelines have been developed [11–14], developing a rigorous evidence base remains problematic. The context in which the decision is made also plays an important part [15]. For example, in secondary care, patients often anticipate the exclusion of secondary pathology and consultants are under pressure to make a diagnosis at the first appointment. These factors result in a wide range of investigation patterns in secondary care with headache investigation rates of up to 60% [16].

Although a number of studies on radiological investigation have been reported from primary care GPs [17–20], there have been no reports from GPs with a special interest in headache. We are unaware of any studies that report the specific reason for imaging or compare these reasons with published guidelines. The aim of our study is to report the reasons GPwSI give for investigation of headache compared with published guidelines and to describe the findings of their investigations.

## Method

14 GPwSIs, all members of the British Association for the Study of Headache (BASH) GPwSI group took part in the study. GPwSIs accepted referrals from their GP colleagues and worked either in a secondary or intermediate care setting. A record was kept of consecutive headache consultations over a 3-month period, whether they were investigated and if so the reason and outcome. Indications for investigation were compared against the BASH recommendations for primary care when brain tumour is suspected [14].

## Results

895 patients were seen, of whom 270 (30.1%) were investigated. Four GPwSIs worked in a secondary care, five in an intermediate care setting and two in a mixed setting (3 were not stated). 59% of the patients were investigated by MRI and the remainder by a CT scan. 15 (5.6%) of investigated patients showed positive findings, a rate of 1.7% of all patients seen.

Table 1 shows the activity breakdown for each GPwSI and outcomes in terms of positive findings. Investigation rates of GPs varied between 12 and 60%. However, only 5 cases (1.9%) were felt to be of definite clinical significance to the headache presentation. Table 2 shows the indications for investigation within the framework defined by the BASH guidelines for primary care and positive findings. 47% of indications were outside the guidance framework. Table 3 expands the reasons other than indicated by the guidance framework that was used. The most common reason for investigation was for reassurance (41.7%).

**Table 1:** Individual GPwSI activity

GPwSI	Number consecutive patients seen in reporting period	Number investigated (%)	Number positive findings of those investigated (%)	MRI/CT of those investigated (%)
1	25	5 (20%)	0	80/20
2	28	8 (29%)	0	63/37
3	43	26 (60%)	2 (7.7%)	18/82
4	29	13 (44%)	1 (7.7%)	62/38
5	57	19 (33%)	3 (15.8%)	95/5
6	59	18 (30%)	2 (11.1%)	28/72
7	64	25 (39%)	0	67/33
8	69	27 (40%)	0	100/0
9	71	10 (14%)	0	40/60
10	84	34 (41%)	2 (5.9%)	100/0
11	150	18 (12%)	4 (22.2%)	94/6
12	76	21 (28%)	1 (4.8%)	100/0
13	58	22 (38%)	0	0/100
14	82	23 (28%)	0	7/93

**Table 2:** Reason for investigation and findings (In some cases 2 or more reasons were listed) within the framework of BASH guidance for GPs when brain tumour is suspected

Indication for investigation within BASH guidance for GPs	Number of indications for investigation (%)	Number of positive findings for each indication (%)	Positive findings
1. Papilledema	1 (0.3%)	1 (100%)	Idiopathic intracranial hypertension
2. Significant alterations in memory, confusion or co-ordination	4 (1.2%)	0	
3. New epileptic seizures	2 (0.6%)	0	
4. New onset cluster headache	7 (2.1%)	0	
5. Headache with a history of cancer elsewhere	11 (3.3%)	0	
6. Headache with abnormal neurological signs or relevant symptoms	29 (8.8%)	0	
7. Headache aggravated by exertion or Valsalva like manoeuvre	27 (8.2%)	6 (22.2%)	Idiopathic intracranial hypertension, subdural, chiari (x3), orbital abnormality
8. Headache associated with vomiting	4 (1.2%)	1 (25.0%)	Sinus thickening
9. Headaches that change significantly	32 (9.7%)	2 (6.3%)	Lesion temporal lobe, aneurysm
10. New headache in a patient over 50 years	43 (13.1)	0	
11. Headache that wake from sleep	11 (3.3%)	0	
12. Confusion	2 (0.6%)	0	
13. Other reason outside of guidance (See Table 3)	156 (47.4%)	6 (3.8%)	(See Table 3)

**Table 3:** Reason for investigation outside of guidance framework and findings

Reason for investigation outside of BASH guidance for GPs	Number investigated (%)	Positive findings
Reassurance	65 (41.7%)	0
Atypical headache	21 (13.5%)	0
Prolonged or complex aura	14 (9.0%)	0
Headache on exertion	7 (4.5%)	0
Orgasmic headache	1 (0.6%)	0
Unilateral tinnitus	5 (3.2%)	0
Cough/valsalva induced headache	6 (3.8%)	0
Thunderclap headache	4 (2.7%)	0
New daily persistent headache	10 (6.4%)	0
Other (not stated)	23 (14.7%)	6 Multiple emboli, infarct [2], aneurysm, glioma, venous sinus thrombosis

## Discussion

We report on the rate and clinical findings of consecutive headache patients seen by 14 general practitioners with a special interest working across a number of settings. 30.16% of patients were investigated. This compares to rates of between 1.2 and 5.3%, where GPs have direct access to neuroradiology investigation [14–18] and up to 60% rates reported to the neurologists [16].

There was a wide range in the number of patients investigated across the practitioners. This possibly reflects different local contexts that include access by GPs to neuroradiology and patient case mix. 15 (5.6%) of the investigated patients showed positive findings, although of those only 5 (1.9%) were felt to be of clinical significance. When GPs have access to investigation, significant abnormalities rates are reported between 2.4 and 1.4% [21] and in secondary care when investigation is clinically selective the yield is 2.1% [16, 22].

We found that the main reason for investigation was reassurance, an important indication reflected in other primary care studies [15, 23]. However, the effects of investigations in terms of reducing anxiety in the longer term produce conflicting findings [24–26]. The identification of incidental pathology, its clinical relevance and the unnecessary anxiety it incurs is well recognised and can be important. We found that 3.7% of investigations showed abnormalities that were not clinically relevant. This compares with population studies of 2.7% [27] and a recent GP study rate of 10% [17].

We conclude that GPs with a special interest in headache investigate at a level that is above GPs, but lower than neurologists whereas yields of significant findings are broadly comparative across all groups. We report that reassurance is the most common cause for investigation. This is difficult to justify on clinical grounds, particularly against a background of limited health care resources and a very low rate of significant findings where there is no clinical suspicion. Direct discussion of patient concerns and the implications of neuroradiological investigation may be more likely to reassure patients than unnecessary tests.

With attempts by many health systems to reduce referral rates to secondary care, access by GPs to neuroradiology is likely to increase. In an area of low yield, our findings may be able to inform revision of guidance on investigation in primary care when patients present with headache.
